# A Chinese Custom

**Published:** 1891-06

**Authors:** 


### A CHINESE CUSTOM.

According to the customs of Chinese society, the wife of the Chinese Minister to the United States will comb her hair up from her forehead, to show that she is married. Her tresses reach to her feet, and so difficult is the task of dressing them that one arrangement lasts several days. For the preservation of the coiffure she lies while asleep on a willow pillow as finely woven as an imported bonnet, shaped like a loaf of baker's bread. The maids dress their back hair in a queue, and arrange a bang one and one half inches deep, from ear to ear. A bit of coquetry is displayed by allowing a single lock to float loosely in front of the face and over the shoulder. The hair of the Chinese girl is never cut, and, as a result of the splendid care bestowed, it grows luxuriantly.

